# Intelligent Detection of Cracks in Metallic Surfaces Using a Waveguide Sensor Loaded with Metamaterial Elements

**DOI:** 10.3390/s150511402

**Published:** 2015-05-15

**Authors:** Abdulbaset Ali, Bing Hu, Omar M. Ramahi

**Affiliations:** 1 Department of Electrical and Computer Engineering, University of Waterloo, 200 University Avenue West, Waterloo, ON N2L 3G1, Canada; E-Mail: abdulbasetali@gmail.com; 2 School of Information and Electronics, Beijing Institute of Technology, Beijing 100081, China; E-Mail: hubing@bit.edu.cn

**Keywords:** artificial intelligence, split-ring resonators, metamaterial, crack detection, waveguide sensors

## Abstract

This work presents a real-life experiment implementing an artificial intelligence model for detecting sub-millimeter cracks in metallic surfaces on a dataset obtained from a waveguide sensor loaded with metamaterial elements. Crack detection using microwave sensors is typically based on human observation of change in the sensor's signal (pattern) depicted on a high-resolution screen of the test equipment. However, as demonstrated in this work, implementing artificial intelligence to classify cracked from non-cracked surfaces has appreciable impacts in terms of sensing sensitivity, cost, and automation. Furthermore, applying artificial intelligence for post-processing the data collected from microwave sensors is a cornerstone for handheld test equipment that can outperform rack equipment with large screens and sophisticated plotting features. The proposed method was tested on a metallic plate with different cracks, and the experimental results showed good crack classification accuracy rates.

## 1. Introduction

There are several well known non-destructive testing (NDT) techniques for detecting surface cracks in metals, such as acoustic emission, eddy current, and magnetic particle testing. However, all have some limitations. For instance, detecting cracks hidden under coatings or under paint is not possible using these methods. Additional limitations apply to cracks filled with different materials such as rust or dirt [1]. Microwave near-field testing techniques have unique advantages over other methods [1]. Unlike other NDT techniques, microwave near-field sensors have the ability to penetrate non-metallic materials and are sensitive to filled or hidden cracks [1–3]. In recent years, artificial engineered electromagnetic materials (metamaterials) have been implemented to create strong localization and enhancement of electrical fields around the sensing element in order to improve the sensitivity and resolution of microwave near-field sensors [4–6]. Metamaterial particles in one-dimensional or two-dimensional arrangements enhance the sensor's sensitivity to detect small anomalies [4,5].

Small signal changes and variations captured by microwave sensors available nowadays are subject to high errors when measured using the human eye. Consequently, the increasing use of microwave near field sensors,necessitates improving their performance,not only at the sensor design level but also at the signal post processing level. Microwave sensors can capture small, complex and hidden information about the material under test. In effect, the collected information (raw datasets) requires advanced signal processing to make accurate decisions. In fact, some decisions are critical, for instance, decisions about the structural health of aircraft fuselages or nuclear reactors. As discussed in [7], machine learning (ML) theories offer a natural framework to address damage detection. Implementing intelligent algorithms for post-process signals collected by microwave near-field sensors can address the challenges associated with small variation or/and information complexity. Incorporating an artificial intelligent (AI) phase within the testing process enhances sensor sensitivity and resolution.

Based on available literature about microwave near-field testing techniques, one can conclude the following: first, research has focused on the integration of machine learning and intelligent signal processing with microwave near-field testing, as in [8–11]. Furthermore, the research that utilized machine learning for microwave near-field testing was mainly directed towards ultra-wide band and dipole antennas for tissue imaging or material characterization but not for detection of cracks in metallic structures. Second, machine learning implementation as in [9–11] has been based on single AI model: artificial neural networks (ANN) or support vector machines (SVM). In the meantime, utilizing advanced machine learning techniques such as boosting, adaptive boosting or other classifier combination techniques mentioned in [12] is expected to improve the accuracy and generalization of the AI model.

This work presents an implementation of an artificial intelligent model for detecting sub-millimeter cracks in metallic surfaces using a waveguide sensor loaded with split-ring resonators. The split-ring resonators, which are constituent elements used in a variety of metamaterial designs, were recently found to enhance the sensitivity of waveguide sensors [6]. The conventional method for sub-millimeter crack detection in metallic surfaces using waveguide sensors consists of observing any frequency shifts in the magnitude of the reflection coefficient (|*S*_11_|) pattern. Additionally, some research has been carried out to change the frequency shift to voltage levels, as in [13], where according to the voltage level, a crack can be detected. With the conventional approach, *i.e.*, shifts in the reflection coefficient, minor shifts are not easily observable or detectable. The importance of employing Al models comes from their ability to detect small variations and to automate the surface scan and crack detection process. Furthermore AI preprocessing methods such as feature normalization can significantly improve crack detection in terms of minimizing the effect of using different standoff distances [14].

In the experimental study presented here, supervised machine learning was used by labeling acquired training datasets from scans of cracked and non-cracked surfaces. Then combined ANNs and SVMs were trained for pattern recognition to classify the surface scans obtained using the sensor. Non-model based classifiers use the whole training data directly for classification of new data without building a model [15]. Therefore, we did not choose classification using non-model based classifiers such as the k-nearest neighbor classifier (KNN). Among model based classifiers, ANNs and SVMs have gained a strong interest because neural networks allow arbitrary nonlinear relations between the independent and dependent variables. In addition, ANNs do not require explicit distributional assumptions (for instance normality) [16]. SVMs are competitive because they build optimal separating boundaries between data sets by solving a constrained quadratic problem [15]. The downside of ANNs and SVMs include the computational resources required during training phase of ANNs [16] and the classification results of SVMs being given with no probability of class membership [15].

Since ANN and SVM classifiers are model based, classification of any new samples is performed by operating the parameters of learned models on the new dataset. For instance, once the ANN has been built (weights have been determined), classification of new data is merely matrix multiplication, a feature that is attractive for hand-held test equipment. The proposed AI models' decisions are made without any need for plotting the captured signals; hence, they can be embedded in infield hand-held crack detection devices.

## 2. Sensor Model and Experimental Setup

**Figure 1:**
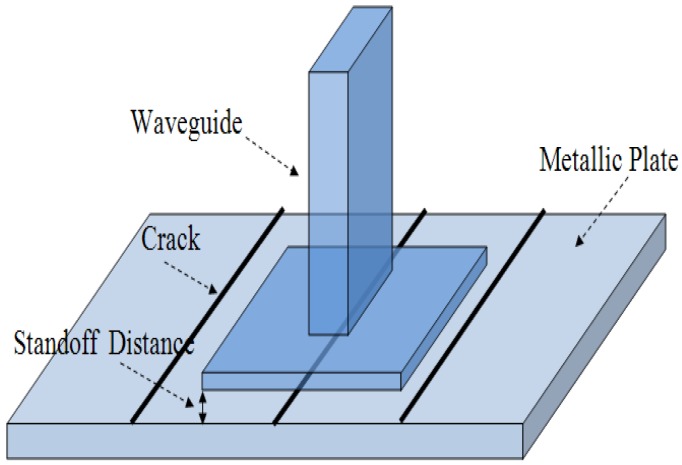
Schematic drawing of a waveguide sensor scanning a metallic plate with surface cracks.

The sensor used in this work for scanning metallic surfaces was an open-ended waveguide probe enhanced with an array of split-ring resonator (SRR) cells [6]. The waveguide is operating at the Ku-band of 12–18 GHz and has a cross section of 15.8 mm by 7.9 mm, with a standard flange with dimensions of 33.30 mm by 33.30 mm. The experimental setup operates by scanning a metallic plate containing 0.5 mm surface cracks ranging in depth from 0.5 mm to 2.25 mm, with increments of 0.25 mm. Figure 1 below shows a diagram of a waveguide sensor scanning a metallic plate with cracks. The sensor is placed 0.5 mm standoff distance, with the long dimension of the waveguide being parallel to the cracks.

By integrating a metamaterial layer between the waveguide and the surface under test the sensor's sensitivity can be enhanced, as presented in [4]. Various shapes for metamaterial cells available in the literature; for instance split rings [17], omega shaped [18], AV-shaped [19] and U-shaped [20] cells. However, for sub-millimeter crack detection a split ring represents an excellent choice due to the high electrical field confinement in the gap. When, utilizing the gap as the sensing element, a major frequency shift was observed if the field in the gap was perturbed due to the existence of a crack [6].

**Figure 2:**
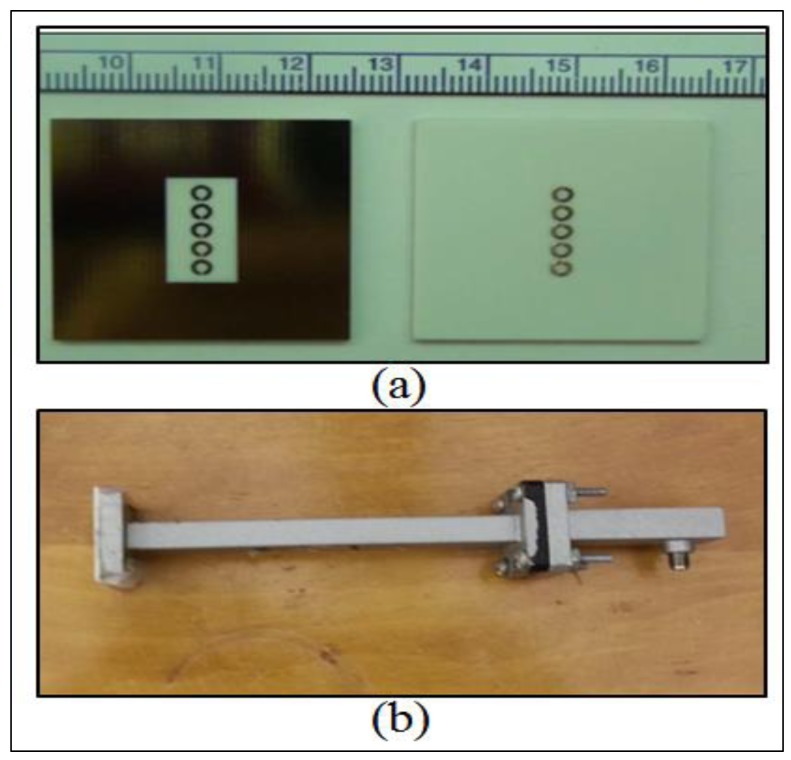
(**a**) Photographs of the front and back views of the split-ring resonator (SRR) array etched on a printed circuit board [6]; (**b**) Photograph of the sensor.

A printed circuit board (PCB) with low loss (Rogers 4003) was used to fabricate the SRRs. Figure 2a displays the front and back views of the PCB patch used at the open end of the waveguide. Figure 2b below shows a photograph of the waveguide sensor.

**Figure 3:**
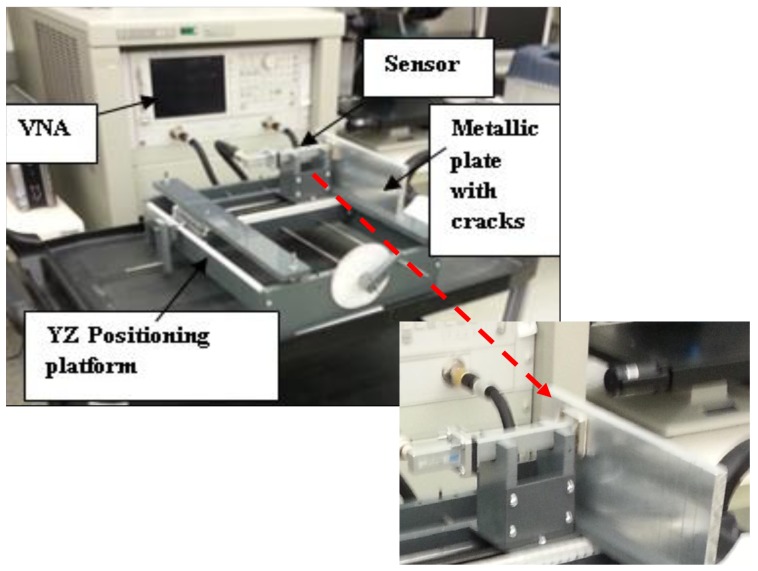
Photograph of the experimental configuration. The scanning along y-axis at 0.5 mm standoff.

In a simplified microwave sensor configuration similar to the one used in this case, the sensor has only one port connected to a vector network analyzer (VNA) as shown in Figure 3, where the sensor is scanning along the y-axis.

**Figure 4:**
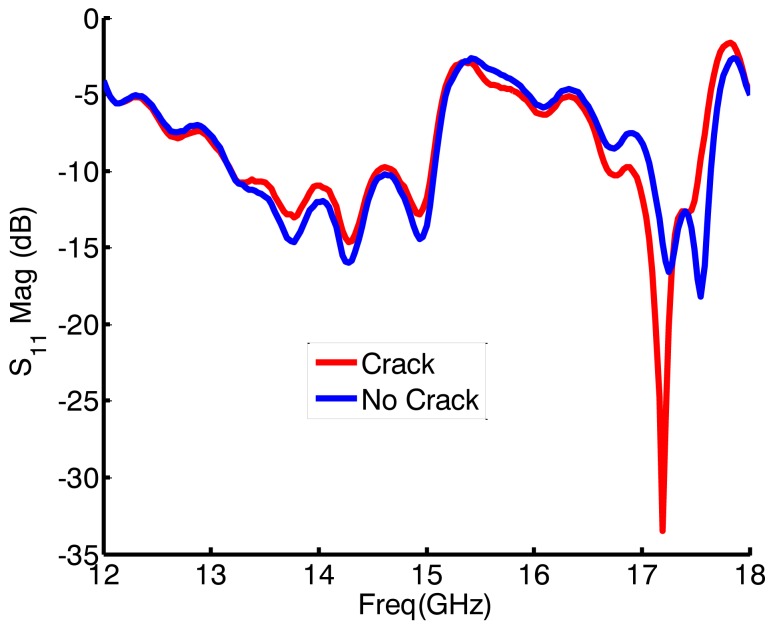
Reflection coefficient magnitude from cracked and no-cracked surfaces at 0.5 mm standoff.

The VNA sends signals to the sensor at different frequencies in a sweep manner and collects the reflected signals (data) from the sensor. The information reflected back is valuable since it reveals details about the sensor's environment. For instance, the reflected data from a non-cracked surface is different from that reflected from a cracked surface. Figure 4 illustrates the reflection coefficient *S*_11_ magnitude over the operating frequency range of the probe from cracked and non-cracked surfaces.

Based on the presence of a crack underneath the sensor, the magnitude of the reflection coefficient (|*S*_11_|) experiences a frequency shift and level change. In other words, the *S*_11_ pattern changes according to the presence or absence of cracks. In this experiment, for each scan, the VNA was swept over a frequency range compatible with that of the sensor (12 to 18 GHz), with increments of 30 MHz. Then the data was collected and saved in an array format to be used later by the AI algorithms.

**Figure 5:**
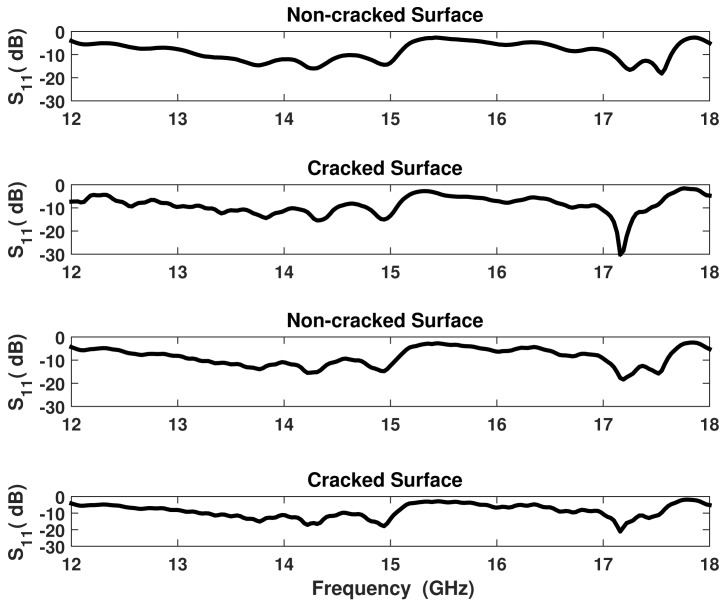
Reflection coefficient magnitude from different cracked and non-cracked surfaces.

In a conventional non-intelligent detection scenario, the reflected data is plotted on the VNA screen, and then trained technicians check for changes in the reflection coefficient pattern, which would indicate the presence of a crack. However, if the crack depth is not large, then observation of a change in the reflection coefficient become a challenge for the human eye. As the cracks in this work vary in depth (from 0.5 mm to 2.25 mm with an increment of 0.25 mm), the reflection coefficient from certain cracked surfaces can hardly be distinguished from that in non-cracked surfaces. Figure 5 shows four measured reflection coefficients. The difference between the first two plots is noticeable, whereas the variation between the last two is subtle and can easily be overlooked.

## 3. Data Processing and PCA Analysis

The data were collected on three different days. On each day, the experiment configuration was rebuilt to ensure full reliability in the repeatability of experimental findings. Then the data was mixed randomly into one dataset, with 415 total samples (scans) and 201 features (frequency points from 12–18 GHz with 30 MHz increments). Additionally, the distribution of the samples was intended to be symmetric (207 samples with a crack and 208 with no crack). In view of the fact that, the first class (cracked scans) and the second class (non-cracked scans) have approximately the same number of samples, the accuracy of the AI model is expected to give a realistic and accurate evaluation of the detection mechanism's performance.

**Figure 6:**
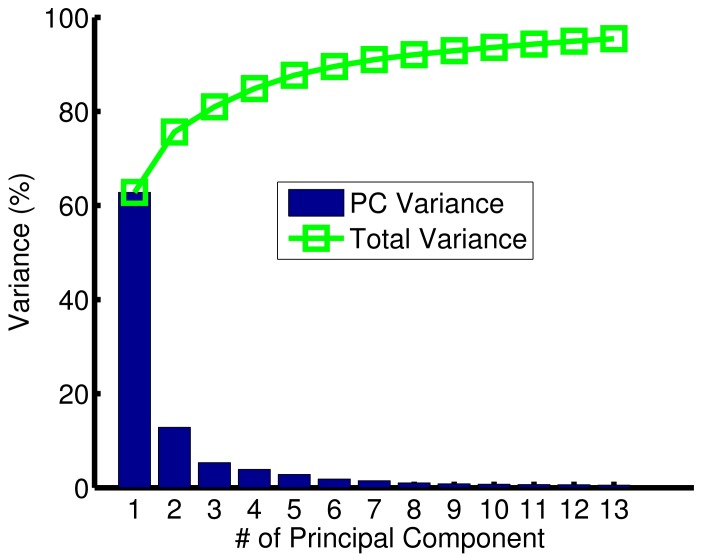
Variance cumulative sum for the first 13 principal components.

Principal Component Analysis (PCA) was used for data pre-procssing. PCA is one of the most widely used techniques for feature extraction and dimensionality reduction to build lower dimension datasets from higher ones. PCA finds a set of the most representative orthogonal projection vectors, where the projected samples retain the most information about original samples with the abilities to remove correlation among variables and enhance the signal to noise ratio [14,21]. Another effective feature extraction method is Independent Component Analysis (ICA), which maps input data onto basis vectors, which are as statistically independent as possible [21]. An important difference between PCA and ICA is related to the number of components used in each methodology. In PCA, this number can be determined by the variance criteria, but in the ICA, there are no criteria for determining how many components represent the dynamic range of the data [22]. Linear Discrimination Analysis (LDA) is another widely used feature extraction method for sensor development [14]. Unlike PCA, LDA requires class information (labels) to find a set of vectors that maximizes the between-class scatter while minimizing the within-class scatter [14,21]. Thus, PCA was implemented in this current study to build datasets of reduced dimensionality by extracting important features according to their variance contribution. PCA was applied after scaling the dataset to zero mean and unity standard deviation (Z-scaling). Figure 6 below shows the PCA results of the first 13 principal components. The PCA analysis shown in Figure 6, makes it clear that the first two principal components contribute more than 75% of the variance. Moreover, the first eight and first thirteen principal components achieve more that 90% and 95%, respectively, of the whole original dataset variance. Data visualization gives a good perspective about the data distribution and can help with selecting the learning algorithm. However, humans can handle only up to three dimensions. Therefore, one of the datasets built using PCA was chosen to be a two-dimensional (2-d).

**Figure 7:**
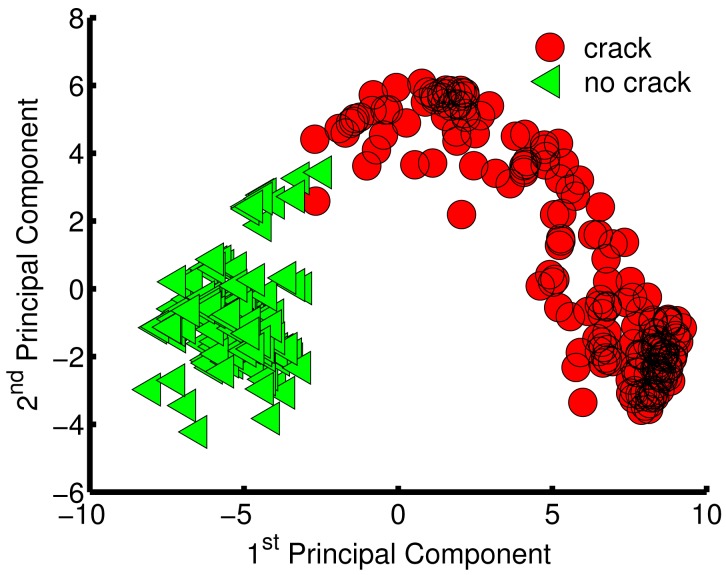
Plot of the first two principal components, which contributed more than 75% of the variance.

To visualize the data distribution in two-dimensional space, the first component was plotted against the second components (Figure 7). The green triangles represent samples/scans for non-cracked surfaces, and the red circles correspond to samples/scans with cracks. One important observation about Figure 7 is that the green triangles are concentrated in one region of the plot, whereas the red circles are widely spread. These concentrations differ because scans for surfaces with no cracks have the same pattern,but scans for cracked surfaces have different patterns as a result of varying crack depths. From the PCA results, three datasets were built in total: Dataset 1: a matrix of 415 samples and 2 features (first two principal components ); Dataset 2: a matrix of 415 samples and 8 features (first eight principal components ); and Dataset 3: a matrix of 415 samples and 13 features (first thirteen principal components that attained more than 95% of the collected data variance).

## 4. Implemented Models

**Figure 8:**
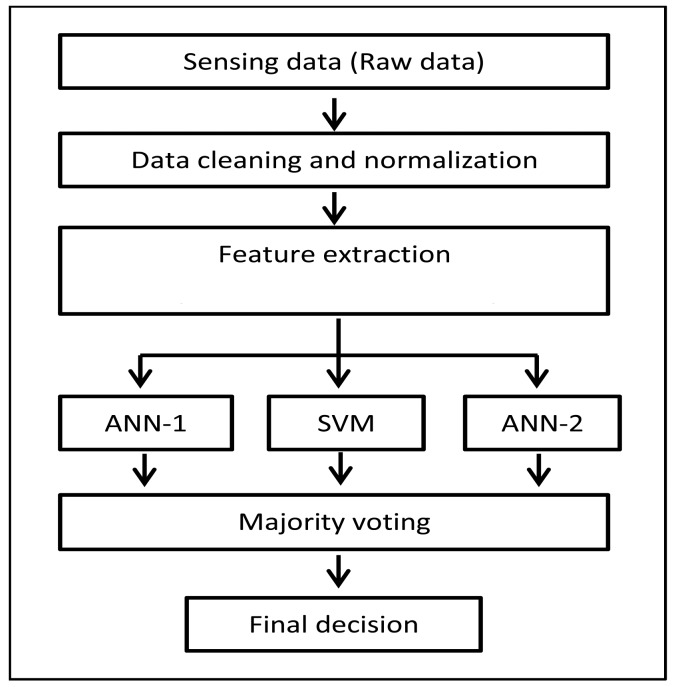
Implemented model architecture. Majority voting combination is used for final decision.

Each feature of the raw data collected by the sensor was normalized to its maximum value. The normalized dataset was then passed to the feature-extraction stage using PCA to generate the three reduced datasets. After feature extraction, the resultant datasets were delivered to the classification stage, where two ANN and one SVM classifiers were implemented to build a combined AI model. Figure 8 provides a flow chart of the data preprocessing and the Al model architecture. An odd number of classifiers was chosen to avoid ties during the combination phase.These classifiers were combined using a parallel manner (Figure 8).

It is important to mention that each dataset had its own AI model because the model parameters were a function of the dataset dimensions. For example, the 2-d dataset needed 2 input units and one bias unit for the ANN input layer; whereas the 8 dimensional (8-d) dataset needed 8 input units and one bias unit.

To maintain a low testing error (out of-sample-error), each dataset was divided into training, validation, and testing subsets, while the model parameters were tuned based on minimizing the validation subset error rate. The sizes of training and validation subsets for each dataset were chosen according to [23]. Table 1 illustrates the size (number of samples) of the training, validation and testing subsets for each dataset.

### 4.1. Artificial Neural Network Classifiers

**Figure 9:**
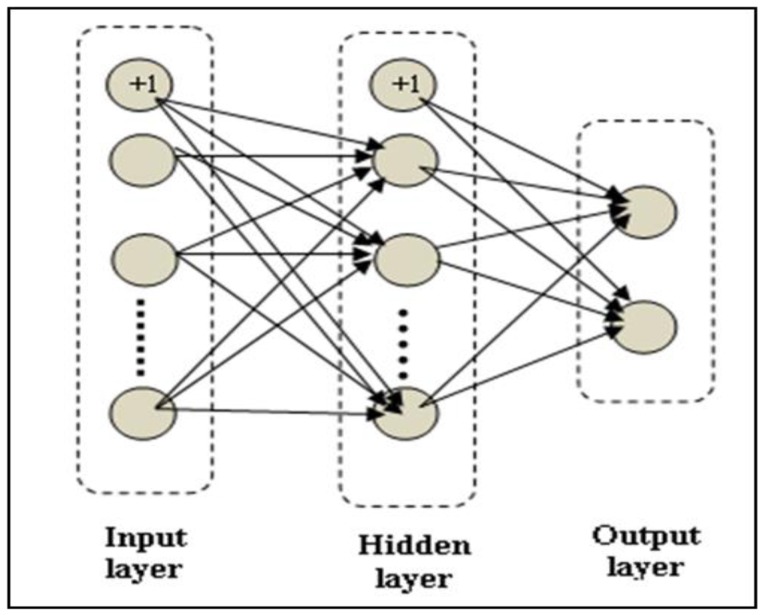
Three-layer neural network with fully connected configuration.

The ANN classifiers implemented in this work were based on a three-layer fully connected configuration, as shown in Figure 9, with a back-propagation algorithm and a log sigmoid function. In fact, different types of activation function used in ANNs, such as linear, step, Gaussian. In this work, a log sigmoid activation function has been selected as it has a convenient derivative (easy to compute from the sigmoid function itself), which is suitable when training networks using back-propagation algorithms [24]. The log sigmoid transfer always limits its output to the range between 0 and 1, as given by

(1) $$g\left(z\right)=\frac{1}{1+e^{-z}}$$

The derivative of the log sigmoid function is given by

(2) $$g^{\prime }\left(z\right)=\frac{1}{1+e^{-z}}*\left(1-\frac{1}{1+e^{-z}}\right)$$

In the literature the back-propagation (BP) algorithm is well studied for ANN training, and it has been used in various applications [25,26]. However, it is subject to local convergence and slowness [27]. On the other hand, particle swarm optimization (PSO) has been gaining interest in recent years and shows good results as in [27]. The PSO algorithm shows faster converge during the initial stages of a global search; nevertheless around the global optimum, the search process is very slow. In contrast, the gradient descend method used in BP tends to achieve faster convergent speed around the global optimum [28]. More recent hybrid algorithms have been reported [28] that combine PSO and BP algorithms to unify the strong global searching ability of the former (PSO) and the strong local searching ability of the latter (BP).

In the current work to help the back propagation algorithm avoid local minima, each ANN classifier was started with random weights for each run. Furthermore, the number of the hidden units was optimized based on the validation error (not on the training error) to avoid over-fitting as much as possible. In addition, a weight decay (regularization parameter) term was included in the algorithm as an additional measure to prevent or minimize over-fitting. The output layer has two units. The first unit gives the probability of a scan belonging to the first class (cracks) and the second unit gives the probability of a scan belonging to the second class (no cracks). At the end, the algorithm assigns the scan to the class with the higher probability.

### 4.2. Support Vector Machine Classifier

The support vector machine (SVM) algorithm has become one of the most effective algorithms for solving problems in classification and regression. An important feature of SVM is that the determination of the model parameters corresponds to a convex optimization problem, thus any local solution is also a global optimum [29]. The SVM is a decision machine and therefore does not provide posterior probabilities.

Considering SVM for classification of a two-class problem using linear models of the form:

(3) $$y\left(x\right)=W^{T}\phi \left(x\right)+b$$

where ϕ(*x*) denotes a fixed feature-space transformation, and b is the bias parameter, Equation (2) can be represented using a dual expression in terms of kernel functions to avoid working explicitly in the feature space (also known as kernel trick; [29]. By implementing support vector machines, the decision boundary between classes has a unique feature, as it is chosen to be the one for which the margin is maximized [12,29]. The implemented SVM classifier was based on a Matlab interface of Libsvm [30]. The SVM classifier achieved high classification accuracy mainly by optimizing the kernel type,degree,and termination criterion tolerance. To have a baseline for the performance of the proposed combined models, a Naive Bayes (NB) classifier was employed. NB and SVM and their variants are often used as baselines in classification tasks, such as in text classification [31]. NB and SVM performance varies significantly depending on the model variant, features, and dataset used [31,32]. Since SVMs was used in the combined models, the NB was considered as the baseline. The average accuracy rate was used as a performance measure by averaging over 1500 runs.

## 5. Results and Discussion

The first model was implemented using Dataset-1 (2-d). Each classifier was optimized individually to maximize the validation accuracy. ANN model optimization was performed by tuning the number of units in the hidden layer and the regularization parameter. Furthermore, to prevent the models from over-fitting, the number of hidden units was kept as small as possible. During SVM optimization, the model showed that linear kernels achieved higher validation accuracy rates than non-linear kernels. The models implemented using Dataset-2 (8-d) and Dataset-3 (13-d) were similarly optimized to maximize validation accuracy. Tables 2–4 give the average training, validation, and testing accuracy rates of each combined model. The standard deviation was also reported to indicate the accuracy rate distribution.

For the Dataset-1 (2-d dataset), the training accuracy rates of the first proposed combined model and the NB were similar, and as high as 99%. However, the combined model slightly out performed its NB model counterpart by 1% during the validation and testing. In the case of the Dataset-2 (8-d dataset), the gap between the training accuracy rates of the second proposed model and the NB model was very small, and a rate of 100% was achievable by the proposed model. However,gaps between the validation and test rates of the second proposed combined model and their NB model equivalent rates were 3.8% and 3.7%, respectively (Table 3).

**Figure 10:**
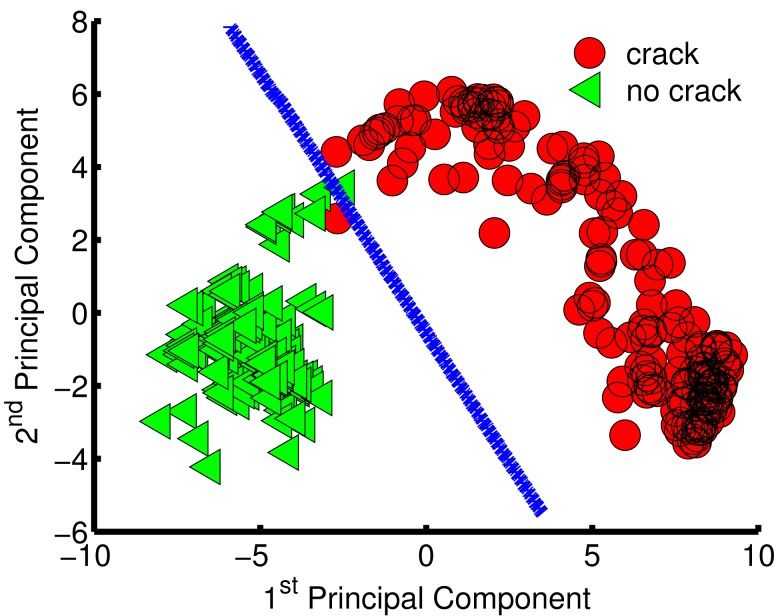
The combined model decision boundary (2-d Principal Component Analysis (PCA) dataset).

Results of the Dataset-3 (13-d dataset), shown in Table 4, revealed that the training accuracy of the third combined model and the NB model were 100% and 99.9%, respectively. Nevertheless, the proposed model achieved higher validation and test rates than its NB equivalent, and the out-performance gaps were increased compared to Dataset-2. More specifically,the difference between the third model validation and test accuracy rates and their NB model equivalents was larger than 5% . Overall,testing of all proposed models revealed a better than 99% accuracy rate for crack classification. A visual explanation of the learned 2-d model is given by Figure 10, where the decision boundary (blue line) is plotted. This boundary separates scans with cracks (red circles) from scans with no cracks (green triangles). The learned model was relatively linear in general. The figure has two misclassified samples. However, avoiding perfect classification (over-fitting) during training is recommended for better out-of-sample generalization.

## 6. Conclusions

This work has demonstrated an implementation of combined artificial intelligent models on different datasets obtained from a microwave waveguide sensor for sub-millimeter crack detection on metallic surfaces. The sensitivity of the waveguide sensor was enhanced with metamaterial particles. Firstly PCA was applied as a feature-extraction technique to obtain a general view of the data in a 2-d space. Additionally, PCA was used to build additional reduced datasets of 8-d and 13-d with more than 90% of the variance of the original dataset. Then, for each dataset a combined AI model was developed that composed of two neural network classifiers and one support vector machine classifier. The results using the three models clearly validate the learning feasibility and the achieved testing accuracy rates were more than 99%. In addition, a base line comparison to a Naive Bayes classifier was implemented as a performance measure.

This paper has demonstrated the practical feasibility of intelligent crack detection in metallic surfaces using AI models and a waveguide sensor. The proposed AI models were able to classify cracks that can easily be overlooked by the human eye. Consequently, this work can be generalized for different types of damage such as, corrosion or precursor pitting. Future work will extend the application of AI models to microwave-based near-field detection of sub-surface anomalies including material and process defects.

Future work will extend the application of AI models to microwaves-based near-field detection of sub-surface anomalies including material and processes defects.

## Figures and Tables

**Table 1:** Size of training ,validation and testing subsets.

**Dataset**	**Training (# of Samples)**	**Validation (# of Samples)**	**Testing (# of Samples)**
2-d	38	8	369
8-d	37	9	369
13-d	36	10	369

**Table 2:** First model using Dataset-1 (2-d) performance profile along with a comparison to Naive Bayes model.

**Model**	**Dataset Features**	**Average Accuracy (Standard Deviation)**		

		**Training**	**Validation**	**Testing**
Combined Model	1st and 2nd (PCA)	99.93% (0.5)	99.30% (2.9)	99.31% (0.5)
NB Model	1st and 2nd (PCA)	99.92% (0.47)	98.18% (4.99)	98.25% (1.64)

**Table 3:** Second model using Dataset-2 (8-d) performance profile along with a comparison to Naive Bayes model.

**Model**	**Dataset Features**	**Average Accuracy (Standard Deviation)**		

		**Training**	**Validation**	**Testing**
Combined Model	First Eight (PCA)	100% (0)	99.50% (2.2)	99.60% (0.65)
NB Model	First Eight (PCA)	99.98% (0.23)	95.77% (7.28)	95.84% (3.2)

**Table 4:** Third model using Dataset-3 (13-d) performance profile along with a comparison to Naive Bayes model.

**Model**	**Dataset Features**	**Average Accuracy (Standard Deviation)**		

		**Training**	**Validation**	**Testing**
Combined Model	First Thirteen (PCA)	100% (0)	99.65% (1.9)	99.62% (0.65)
NB Model	First Thirteen (PCA)	99.98% (2.1)	94.3% (8.21)	94.48% (3.78)
